# Resident Training in Minimally Invasive Spine Surgery: A Scoping Review

**DOI:** 10.3390/brainsci15090936

**Published:** 2025-08-28

**Authors:** Michael C. Oblich, James G. Lyman, Rishi Jain, Dillan Prasad, Sharbel Romanos, Nader Dahdaleh, Najib E. El Tecle, Christopher S. Ahuja

**Affiliations:** Department of Neurological Surgery, Northwestern University Feinberg School of Medicine, 676 N. St. Clair Street, Suite 2210, Chicago, IL 60611, USA; michael.oblich@northwestern.edu (M.C.O.); james.lymaniii@northwestern.edu (J.G.L.); rishi.jain@northwestern.edu (R.J.); dillan@northwestern.edu (D.P.); sharbel.romanos@northwestern.edu (S.R.); nader.dahdaleh@northwestern.edu (N.D.); najib.eltecle@nm.org (N.E.E.T.)

**Keywords:** minimally invasive spine surgery, resident education, simulation training, neurosurgery, orthopedic surgery

## Abstract

**Background/Objectives**: Minimally invasive spine surgery (MISS) is complex and requires proficiency with a variety of technological and robotic modalities. Acquiring these skills is a long and involved process, often with a steep learning curve. This paper seeks to characterize the state of MISS training in neurosurgical and orthopedic residency programs, focusing on their effectiveness at minimizing substantial learning curves in the field, as well as highlighting potential areas for future growth. **Methods**: We conducted a scoping review of the PubMed, Scopus, and Embase databases utilizing the PRISMA extension for scoping reviews. **Results**: Of the 100 studies initially identified, 16 were included in our final analysis. MISS training types could be broadly grouped into four categories: virtual simulation (including AR and VR), physical models, hybrid didactic and simulation, and mentored training. Training with these modalities led to improvements in resident performance across multiple different MISS techniques, including percutaneous pedicle screw fixation, MIS dural repair, MIS-TLIF, MIS-LLIF, MIS-ULBD, microscopic discectomy/disk herniation repair, percutaneous needle placement, and surgical navigation. Specific improvements included reduced error rate, operation time, and fluoroscopy exposure, as well as increased procedural knowledge, accuracy, and confidence. **Conclusions**: The incorporation of MISS training modalities in spine surgery residency leads to increases in simulated performance and could serve as a means of overcoming significant learning curves in the field.

## 1. Introduction

Despite their potential, the adoption of minimally invasive surgical (MIS) techniques in spine surgery is often hindered by their steep learning curve [1,2,3]. Junior doctors exploring MIS in training or those practicing the techniques for the first time often cite several concerns, including decreased exposure of anatomical landmarks [4], unfamiliarity with endoscopic imaging orientation [5], and suboptimal trajectory during approach, leading to increased intraoperative readjustment and higher complication rates at early stages of the learning process [5,6]. Downstream, these intraoperative complications can increase operative time [7], readmission rates [8], and length of hospitalization [9].

Previous studies have described the minimally invasive spine surgery (MISS) learning curve across multiple different minimally invasive procedures and indications, including MIS-lumbar discectomy [9,10], microendoscopic decompression for spinal canal stenosis [11], and MIS-TLIF [8,12,13]. The considerable time and effort required to overcome these learning curves can create barriers to surgeon uptake of MISS, especially among surgeons accustomed to performing open techniques [3,5]. It is estimated that attending surgeons can achieve 50% proficiency (proficiency was defined differently depending on the study. In general, it was a composite metric factoring in operation time, complication rate, and procedural outcome) with a particular MISS procedure after approximately 12 cases, while 90% proficiency requires 25 to 40 operations [3]. This barrier to uptake is suboptimal, as minimally invasive techniques have consistently been associated with improved patient outcomes following spinal surgery as compared to traditional open methods, with benefits including reduced surgical site infections, shorter length of hospital stay, and reduced intraoperative blood loss [14]. Additionally, a 2023 study by Passias and colleagues found MIS techniques to be associated with lower intra- and perioperative complication rates, reduced need for reoperation, and improvements in 1- and 2-year postoperative disability scores in patients with increased frailty [15]. This suggests that MISS techniques may allow for expanded treatment options for patients who have a decreased tolerance for large open procedures. Thus, despite notable barriers to its uptake, the significant benefits provided by MISS necessitate its continued implementation.

The learning curve in MISS is surmountable given appropriate time and resources dedicated to practicing the respective procedures. Regardless of etiology, MISS complications are disproportionately associated with initial inexperience and fall off drastically once sufficient experience is gained. In a 2014 systematic review investigating the uptake of five different MISS techniques by attending spine surgeons, Sclafani et al. found an overall complication rate of 11%, but no complications after surgeons had completed at least 30 cases [5]. Thus, the evidence suggests that with sufficient procedural volume the deleterious impact of MISS learning curves can be overcome.

While substantial experience is necessary to achieve competency with MISS techniques, the question of how this experience can and should be gained is still largely unanswered. Much of the literature investigating the MISS learning curve is focused on attending surgeons previously trained in open techniques incorporating minimally invasive techniques into their practice. While this is informative, the increased rate of MISS utilization in today’s landscape means that neurosurgical and orthopedic surgery residents have increased exposure to these techniques. Evidence from other specialties suggests that early training with minimally invasive techniques during, and even before, residency reduces learning curves and improves outcomes [16,17,18]. Thus, through the structured incorporation of MIS earlier in the training process, spine surgery programs may be able to “shift” the learning curve years earlier, thereby reducing patient risks and decreasing the burden on healthcare systems. The objective of our study is to comprehensively explore the integration of MIS techniques into neurosurgical and orthopedic spine surgery training, with a focus on identifying MISS training modalities, determining their efficacy and practicality, and exploring potential applications.

## 2. Materials and Methods

### 2.1. Literature Search Strategy

We performed electronic literature searches in the PubMed, Embase, and Scopus databases from inception through to February 2025. The searches combined Medical Subject Headings (MeSH) terms and free-text keywords relating to minimally invasive spine surgery, spine surgery training, learning curves, simulation, and surgical education in orthopedic and neurosurgical residency programs. Additional articles were identified by manually screening references of relevant publications. Full search terms for each database are listed in Supplementary Table S1.

As this was a scoping review, we did not register our protocols publicly before data extraction began. However, we strictly followed the protocols outlined by the PRISMA extension for scoping reviews (PRISMA-ScR).

### 2.2. Inclusion Criteria and Screening Process

**Figure 1:**
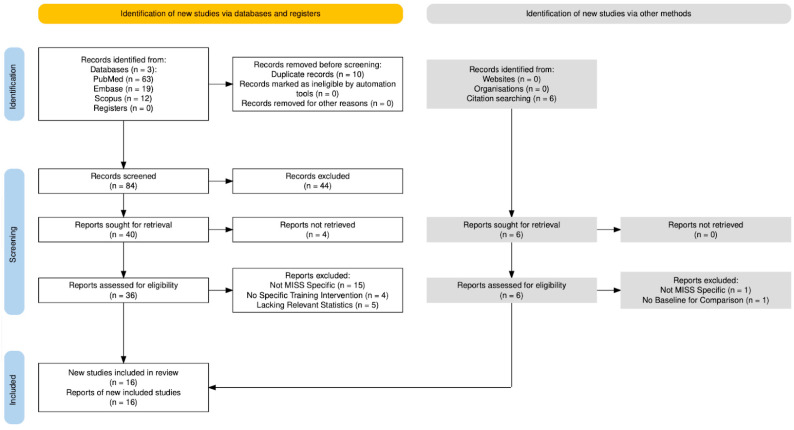
PRISMA-ScR workflow for article screening. Of the 100 articles initially identified, 16 were retained to be included in the final analysis.

The screening process was conducted using the Preferred Reporting Items for Systematic Reviews and Meta-Analyses extension for scoping reviews (PRISMA-ScR) (Figure 1). We included peer-reviewed articles in English that discussed (1) minimally invasive spine surgery techniques, (2) the learning curve and/or skills acquisition relevant to MISS, and (3) training paradigms, simulation models, or curricula for spine surgery trainees such as medical students, residents, and spine fellows. For the purposes of this review, MISS was defined as any microscopic, endoscopic, or robotic spinal procedure conducted by orthopedic surgeons or neurosurgeons via small incisions with tissue-sparing, often image-guided, techniques. Studies focusing solely on open techniques, lacking specific reference to training, or not relating to spine surgery were excluded. Article types included review articles, randomized controlled trials, cohort and case–control studies, case series, conference proceedings, and consensus or position statements. Editorials, commentaries, and articles published more than 25 years prior were excluded. Each identified article’s title, abstract, and full text were screened for relevance. After initial screening by two independent reviewers (MO, JL), conflicts were resolved by discussion with a third reviewer (RJ). Articles meeting the criteria were retained and the final reference list was then generated. Given the diverse nature of studies included in this scoping review, we did not perform a formal risk-of-bias or quality scoring process. However, all reviewed sources were appraised qualitatively for methodological rigor, clarity in outcome reporting, and relevance to the topic of MISS training. Where possible, we prioritized higher-level evidence.

### 2.3. Data Extraction and Synthesis

We extracted data pertaining to the type of MISS technique(s) studied, the type of training approach implemented, and relevant outcomes from each educational model. Because of the heterogeneity in study designs, outcomes, and MISS techniques described, quantitative synthesis or meta-analysis was not feasible. Instead, we performed a descriptive synthesis to outline common themes, identify benefits and limitations in current training paradigms, and propose recommendations to improve training and advance the field. An AI tool (ChatGPT, Version 4o) was used to assist in proofreading and formatting only. All data extraction, synthesis, and manuscript preparation was completed by the authors.

## 3. Results

### 3.1. Study Selection

A total of 94 studies were identified via a search of PubMed, Embase, and Scopus databases. An additional six studies were identified from the citations of relevant literature, for a total of 100 studies. After the removal of duplicates, 90 studies were included in an initial title and abstract screening. Following abstract review, 46 studies were assessed for eligibility using the inclusion/exclusion criteria outlined above. Of these studies, complete manuscripts could not be retrieved for four. Of the 42 full reports assessed for eligibility, 26 were excluded for lack of MISS specificity (n = 16), lack of relevant statistics (n = 5), no specific training intervention discussed (n = 4), and lack of baseline for comparison (n = 1). This left 16 studies remaining for data extraction and inclusion in our review. A full PRISMA extension for scoping reviews workflow detailing the screening process is shown in Figure 1.

### 3.2. Study Trends and Key Findings

The publication years of the 16 included studies ranged from 2009 to 2025, with five (31.25%) from the last five years. Nine (56.25%) studies were prospective pre-post intervention, two (12.5%) were randomized control trials, and two (12.5%) were prospective cohort. The remaining three consisted of a prospective within-subjects controlled study, a prospective comparative study, and an observational study. Study sizes ranged from 5 to 102 participants, with a median sample size of 10.

The most common MISS technique discussed was percutaneous pedicle screw insertion, serving as the focus of half (8/16) of the included literature. Other techniques highlighted included minimally invasive surgical approaches for lateral lumbar interbody fusion (MIS-LLIF), transforaminal lumbar interbody fusion (MIS-TLIF), unilateral laminectomy for bilateral decompression (MIS-ULBD), lumbar discectomy, dural repair, and lumbar disk herniation repair, as well as percutaneous needle placement and surgical navigation.

Training types could be grouped into four major categories based on shared characteristics. They include virtual simulation (6/17), physical models (5/17), hybrid didactic and simulation (4/17). The median sample sizes across these training categories were relatively homogenous, ranging from 8 to 12 participants. Mentored training had smallest number of included studies (2/17), although the median sample size was the largest (n = 59.5). One study focused on multiple different training modalities, which accounts for the 17 different training methods for only 16 studies. The key findings from each study can be found in Table 1.

### 3.3. Breakdown by Training Modality

#### 3.3.1. Virtual Simulation

The most common MISS training paradigm identified was virtual simulation, including a mix of pure computer-run training programs, virtual reality and augmented reality platforms, and multimodal systems that incorporate VR/AR. While grouped together under the heading of “virtual simulation”, computer programs, VR, and AR are distinct modalities with unique features. VR and computer-based systems rely completely on a virtually rendered models, with total separation from the physical world. This allows for the simulation of multiple procedures or techniques using the same system, but limits real-world haptic feedback. AR-based systems also rely on virtual rendering, but digital images are overlayed on the real world. This allows for real-time feedback from both the digital and physical environment [35]. While there were multiple different methods of virtual simulation training, certain key themes emerged from our analysis. Increased fixation accuracy during simulated MISS procedures, as well as reduction in operation time and amount of fluoroscopy exposure, were noted as primary outcomes of training with purely VR or computer-run simulation training [19,24]. Training systems that incorporated primarily augmented reality or multimodal AR approaches found similar results, with the primary outcome being reduced use of fluoroscopy necessary to achieve satisfactory results [29,34]. Finally, a recent study found that the supplementary incorporation of AR when training on a high-fidelity spine model significantly reduced self-reported mental demand and difficulty maintaining optimal surgical performance [23].

#### 3.3.2. Physical Models

Another common training methodology identified was the use of physical models. This includes a wide range of unique systems, including 3D-printed spinal models, human and animal-based cadaveric models, and high-fidelity synthetic models. As was the case with virtual simulation, residents demonstrated significant decreases in mean procedural time after implementation [20,22,33]. Akbulut et al. found that by the fourth practice attempt on their 3D-printed model, the mean time to successfully complete a simulated MIS lumbar discectomy was reduced by more than three times [20]. Buchanan et al. demonstrated a similar significant reduction in operative time for MIS dural repair after practice using a perfusion-based cadaveric model [22]. They found that, regardless of post-graduate year, trainees were able to successfully complete a sturdy dural closure on the model via MISS techniques and by their final attempt could do so in nearly half their initial time. Melcher et al. also noted a progressive decrease in average procedural time, as well as the number of skipped steps and surgical errors over three trials performing MIS-ULBD on a high-fidelity spinal model [33]. The authors also identified decreased knowledge gaps as well as increases in overall surgical proficiency, efficiency, and handling of instruments, especially in junior residents. Finally, Walker et al. found that both junior and senior residents reported increased confidence in their ability to perform pedicle screw insertion and MIS laminectomy procedures, respectively, after conducting simulated procedures on an animal cadaver-based spinal model [26].

#### 3.3.3. Hybrid (Didactic + Simulation)

Hybrid didactic and simulation-based training was the third most common MISS teaching modality implemented in the literature, combining didactic explanatory modules with hands-on, simulation-based techniques. The training courses shared similar characteristics, but each had slightly different implementation and outcomes. Harrop et al. and Chitale et al. found that the addition of a two-hour didactic course before training on a physical model improved resident performance in both written and technical skills evaluations for MIS-PCDF and percutaneous pedicle screw insertion, respectively [21,25]. The latter study found the greatest improvements in residents’ ability to use fluoroscopic and CT guidance [21]. Gardeck et al. incorporated a MIS pedicle screw fixation curriculum which included both a lecture component on surgical navigation and training sessions with a synthetic spinal model. All 15 residents who participated demonstrated significant improvements from first to second sessions in subjective measures of navigated screw placement, while 14 of 15 demonstrated significant improvements in objective measures, including amount of time per screw placement, regardless of prior familiarity with instrumentation [32]. Finally, Sundar et al. found that the addition of a spinal navigation educational session to training using cadaveric or SawBones spinal models led to a statistically significant decrease in percutaneous pedicle screw insertion surgical error, as compared to a control group without the educational training. Additionally, those who participated in the hybrid training had significantly improved accuracy of screw placement in cervical, thoracic, and lumbar spinal regions [31].

#### 3.3.4. Mentored Training

Mentored, apprenticeship-style instruction has traditionally been the most common surgical training modality [36,37]. For the purposes of this review, mentored training encompassed both resident involvement in actual MIS procedures, as well as expert surgeon guidance during model-based simulation. Our literature search revealed two papers focusing on training modalities which met these characteristics. First, Stienen et al. found no significant differences in intra- or postoperative outcomes between resident-involved and non-resident-involved microscopic lumbar herniation repair procedures [27]. Second, in a randomized control trial, Kirkpatrick found that residents practicing percutaneous pedicle screw insertion on MIS spinal models showed significantly greater overall improvement and significantly reduced error rate when a skilled mentor was present to instruct them [30].

## 4. Discussion

Our scoping review identified four primary MISS training modalities: virtual simulation, physical models, hybrid didactic/simulation, and mentored training. While some included studies discussed training at the medical student and fellowship levels, our review focused primarily on training for orthopedic and neurosurgery residents. It should also be noted that experienced surgeons may contend with learning curves when performing MISS procedures. However, attending physicians are not governed within the same training structure as residents and may reasonably perform a novel MISS technique, given patient consent, without the same level of oversight. Thus, the outcomes discussed throughout this review are most applicable to trainees at the residency level. Specific training methods and curricula differed depending on the institution and type of minimally invasive technique, but they all improved resident performance in at least one (and often multiple) aspects of the MISS skills being learned. Cumulatively, the literature is in favor of the early adoption of simulation-based training for the development of orthopedic and neurosurgical residents.

Multiple different strengths and weaknesses were identified based on each specific training type. Virtual simulators are excellent at providing trainees with real-time procedural guidance and feedback [28]. As a result, virtual simulation can be particularly beneficial for training in MISS, which relies almost exclusively on access through narrow surgical windows requiring precise navigational trajectories. These systems have the capability to overlay anatomic landmarks, essentially allowing residents to “see through” virtual or real models they are practicing on. They also provide trainees with the opportunity to receive instant feedback on procedural accuracy. Frequent practice with these models familiarizes residents with the correct anatomy and trajectory for a minimally invasive surgical approach, allowing for more efficient operations with less fluoroscopy exposure. Virtual simulation training modalities also have the added benefits of ease of access and essentially unlimited opportunities for independent resident practice, as the systems do not require access to operating rooms with real patients or significant oversight from attending surgeons. Going forward, virtual simulation could serve as a way for residents to quickly and independently gain valuable practice in MISS, resulting in more accurate and confident surgical approaches while limiting fluoroscopy exposure for patients once the time comes for involvement in real procedures.

A potential drawback of VR based simulation is the significant costs associated with the acquisition of commercially available systems [38,39]. However, Ghaednia et al. found that head-mounted VR devices (e.g., Occulus Rift), which are significantly cheaper than full VR surgical systems, could still be an effective educational tool for spine surgery [40]. Although no included studies incorporated a comprehensive economic analysis of VR-based training specific to MISS, studies from other surgical fields have shown it to be cost-effective despite high initial overhead expenses [41,42]. Thus, while cost could be a potential limiting factor to the implementation of VR-based simulation training in MISS, it should not be considered an absolute barrier. Further cost–benefit analysis within the field is needed before definitive conclusions can be drawn.

As identified by Ryu et al., the greatest strength of physical MISS models is the provision of more realistic haptic feedback and the ability to practice using real surgical instruments and equipment [28]. Training on physical models allows residents to experience the sensation of performing operations on realistic, often life-sized “patients”. As a result, trainees gain tangible insights into how physical bone, tissues, and vasculature will respond during minimally invasive surgery. This stands in contrast to virtual simulators which often attempt to emulate physical sensation via artificial haptic feedback. Perhaps the most important advantage of physical models is the ability of residents to gain familiarity with real surgical tools and equipment. Knowing how to operate and contend with real-life limitations and complications of various surgical instruments, including hardware and navigational systems is a challenging aspect of MISS training and is one of the primary drivers behind its difficult learning curve. Early exposure and practice with MISS tools and equipment via physical models can help improve resident proficiency and confidence in their utilization. In theory, this could lead to less time spent focusing on equipment operation and more time dedicated to learning procedural mastery during resident teaching cases in the operating room.

Two limiting factors when it comes to physical models are cost and lack of case variety. Cadaveric and animal-based simulation models are expensive to maintain and require significant upkeep [43]. While non-cadaveric “phantom” synthetic models can be produced with relative ease and at low expense, they are time-consuming to manufacture [44]. Considering that each synthetic model must be tailored to a specific surgical scenario or scenarios, it could become impractical to develop a comprehensive MISS simulation curriculum using only fabricated models. Programs could consider implementing a combinatorial approach with both cadaveric/animal and synthetic MISS training modalities in order to gain the significant benefits of physical models in a more practical and cost-effective manner.

The strength of hybrid didactic and simulation training is providing an opportunity for conceptual “classroom” learning before reinforcement on physical models. Instructing residents on the fundamentals of MISS techniques (such as fluoroscopic navigation) helps develop a stronger understanding of fundamental principles before they are solidified via hands-on training with a physical model. In addition to conceptual knowledge, hybrid instruction also provides residents with the same advantages of physical model use, namely increased familiarity with surgical equipment. However, this also means that hybrid training models are subject to the same cost and variety limitations as physical models.

While it was the least discussed MISS training subtype identified in our scoping literature review, mentored training is undoubtedly one of the most common techniques in surgical education as a whole [27,37]. This likely has to do with the fact that resident involvement in teaching cases and mentored cadaveric dissection has been the only real type of surgical training available throughout much of medical history. The primary benefits of this teaching avenue are the ability to practice MISS techniques on real tissues and, in the case of teaching cases, in a live, real-life scenario. Live surgical experience is invaluable, as even the most detailed models and simulators can never fully replicate the nuances and intricacies of a real patient in the operating room. Training through real surgical experience therefore does not raise the same concerns over external validity (i.e., improvements in the simulator that do not translate to real surgical skill) as simulation-based models. Additionally, the “on-the-job” instruction provided by expert attending surgeons and the experience gained working with the surgical team can be extremely valuable for residents learning the workflow of minimally invasive spine surgery. It should be noted that this form of training is primarily applicable to residents and fellows, as medical student involvement in procedures is generally limited to observation and basic tasks such as retraction and suction. While some studies, including one referenced in this paper, have demonstrated no significant risk to patients due to resident involvement in MISS teaching cases, there are other lingering concerns with this training modality. The involvement of a trainee during any procedure adds another potential avenue for complications which must be accounted for by the attending surgeon and other members of the surgical team. Another potential drawback of this training modality is that it depends on the availability of surgeon mentors and the number of MISS cases open for resident involvement. Additionally, priority for involvement in procedures is often given to fellows and senior residents. Finally, even when trainees are able to scrub into MISS cases, it can be difficult to be actively involved in the procedure due to the inherently limited operative window. Thus, while surgeon mentorship and resident involvement in teaching cases are effective tools for MISS training, they should be employed as supplements to other training modalities not as affected by these limiting factors.

The various MISS training modalities highlighted in this review could be used to address the shortcomings of mentored training and help promote the efficient use of valuable mentored training time. Virtual simulation promotes a greater understanding of surgical trajectories and increased efficiency with fluoroscopic navigation. Training with physical models allows residents to gain familiarity with surgical instruments and equipment in a realistic, but safe and controlled, environment. Hybrid didactic training fosters improved conceptual understanding of minimally invasive techniques before reinforcement on physical models. All three of these training modalities offer opportunities for residents to learn and improve their ability to execute the fundamental elements of MISS procedures in a structured, safe, and accessible manner. Early, deliberate implementation of these simulation-based training types during neurosurgery and orthopedic residencies allows for greater focus on learning operating room dynamics, finer procedural details, and case-specific nuances during live teaching cases. Having already mastered the basics using virtual, physical, or hybrid training models, residents are able to approach MISS procedures with increased efficiency, proficiency, and confidence. This, in turn, may allow for even greater opportunities for resident involvement in more complex aspects of MISS procedures, under the guidance of experienced attending surgeons. Thus, while simulation-based training is not a substitute for involvement in live procedures, its early implementation may serve as a potentiator of resident education in minimally invasive spine surgery and, in turn, help shift the burden of significant MISS learning curves away from the operating room.

Despite strong evidence for the implementation of early MISS training and the existence of multiple different validated simulation modalities, there is a lack of a universal, standardized MISS residency curriculum, complete with validation standards and benchmarks. This stands in contrast to other surgical specialties that heavily employ minimally invasive techniques and surgical robotics with great success, such as General Surgery, Gastroenterology, Otolaryngology, Urology, and Gynecology, which all have established curricular parameters with incorporated simulation [45,46,47]. This relative deficit may be partly due to economic, regulatory, and cultural challenges constraining broader integration of robotics within spine surgery [48]. De Win et al. found that the incorporation of just 18 h of intensive laparoscopic simulation training in the final year of medical school markedly increased the performance of general surgery residents during laparoscopic cholecystectomies [16]. At 6 months into training, the odds of adverse events during surgery were 4.5 and 3.9 times lower, respectively, for residents who had received early intensive training as compared with those receiving none or only intermittent additional training [16]. Walliczek et al. identified total repetition frequency as the key factor driving gains in specific outcomes, including overall technical performance, time to complete, and economy of motion with the DaVinci surgical robot system [17]. This suggests that more time allocated to practicing with minimally invasive surgical technology could lead to improved uptake of MIS skills among novices. It has also been demonstrated that a 4-week training program dedicated to the da Vinci surgical robotic system significantly improved the technical performance of novices, both on previously attempted and novel tasks [18]. Thus, while our literature search did not find any eligible studies on robot-assisted MISS training, results from other specialties suggest that increased familiarity with robotic MIS systems through dedicated training could improve surgical performance on previously attempted tasks while also ameliorating the effects of the learning curve when developing proficiency with new MIS procedures.

Professional societies have made efforts to standardize and optimize the use of simulation-based training in residency programs. The American College of Surgeons (ACS) and Association of Program Directors in Surgery (APDS) developed a three-stage curriculum emphasizing proficiency-based, competency-driven integration of surgical skills. Assessment with objective tools, such as surgical simulators, sets the pace for advancement rather than module completion [49]. However, one study found that the incorporation of these curricular guidelines in orthopedic surgery and neurological surgery residency programs is relatively low, at 63% and 54%, respectively [50]. These relatively low adoption rates of the ACS/APDS simulation-based training curriculum may reflect a need for specialty-specific curriculum for orthopedic and neurological surgery training.

Synthesizing from our review, we propose a framework for MISS training that incorporates stepwise, structured implementation of training modalities throughout orthopedic and neurosurgical residency. During the first two years of training, residents should gain initial exposure to common MISS procedures and the fundamental skills of MISS. The implementation of virtual simulation training sessions, including VR and AR, would allow junior residents to develop early anatomical familiarity and practice trajectory planning, two major contributors to the MISS learning curve. Alongside this initial simulated exposure, hybrid didactic training sessions should be introduced to reinforce critical skills and develop necessary background knowledge (e.g., indications for MISS versus open techniques). Once initial competency benchmarks for basic MISS techniques are achieved in virtual and didactic training, physical models should be introduced. This transition should ideally occur in the second or third year of training and occur alongside continued practice with virtual models and didactic sessions. Physical models should be introduced in a stepwise fashion, beginning with simple models for basic skills (e.g., 3D printed model for pedicle screw fixation) and eventually progressing to more complex cadaveric and high-fidelity synthetic models. Whenever possible, training sessions with physical models should be mentored by a senior resident, fellow, or (ideally) an attending spine surgeon. Having developed foundational knowledge, skills, and familiarity with MISS procedures through model-based training, residents may now be gradually introduced to mentored training in the operating room. In the later years of training, resident involvement in MISS procedures should increase with graduated autonomy, although always under direct supervision from an attending surgeon mentor. At this stage, MISS training programs should employ validated assessment tools such as the objective structured assessment of technical skills (OSATS) to ensure residents achieve proficiency through structured, evidence-based assessment rather than time or case volume alone [51]. Early, structured incorporation of simulation-based MISS training modalities, extensive mentored training, and objective, benchmark-driven progression may allow trainees to progress farther along the learning curve during residency and ultimately be better equipped to conduct MISS procedures upon graduation.

Collectively, the current body of literature from both spine surgery and other minimally invasive surgical specialties supports the idea of early and frequent implementation of MIS education in surgery training. The relatively small number and design heterogeneity of included studies precluded quantitative synthesis or direct comparison of training effects across modalities. Additionally, small sample sizes (median n = 10), single-center designs, and reliance on non-randomized methods for a majority of studies may reduce the generalizability of conclusions. Despite these limitations, our scoping review highlights the need for a more thorough investigation into the long-term efficacy of simulation-based training modalities in minimally invasive spine surgery training, as well as the need for standardized MISS training and assessment protocols across neurosurgery and orthopedic residency programs. Potential avenues for future research include multicenter implementation studies of training models, curricular development consensus studies, and validation of simulation results against clinical endpoints. Likewise, a thorough cost–benefit analysis comparing various simulation-based MISS training modalities is warranted to determine the practicality of their implementation across different settings.

## 5. Conclusions

Minimally invasive spine surgery (MISS) training has benefited from ever-expanding simulation modalities, including computer-based platforms, high-fidelity synthetic models, and cadaveric courses. Collectively, these approaches have been shown to accelerate skill acquisition and confidence in the early stages of residency. Multiple studies demonstrate reductions in operative time, enhanced pedicle screw placement accuracy, and better overall technical performance among trainees who undergo structured simulation. Notably, integrating didactic components, structured expert feedback, and repeated practice appears most conducive to sustainable skill development. The early introduction of MISS training techniques can serve as a catalyst for resident education and minimize reliance on intraoperative experience. While these training paradigms cannot replace real-world operative mentorship, they offer an adjunct that can help shift the learning curve to a time when direct supervision is more readily available.

Despite the promising advancements in simulation and technology-enhanced training, the existing literature is limited by heterogeneous study designs and modest sample sizes. Current research into MISS training has significant gaps, including a lack of long-term efficacy and cost-effectiveness studies. Additionally, our review found no standardized training benchmarks for MISS. For these reasons and others, there is an uneven adoption of standardized MISS curricula across neurosurgical and orthopedic residency programs. Yet, given the proven early-stage benefits, there is a need for widespread, structured implementation akin to the systematic approaches employed by other minimally invasive surgical specialties. As proposed, programs should consider embedding frequent simulation sessions, targeted didactic modules, and proficiency-based progression early into their existing educational frameworks.

Future research and multi-center collaborations would help define best practices, validate long-term efficacy, and clarify how to most efficiently optimize resources across training environments. Ultimately, a concerted effort to incorporate comprehensive MISS education early in residency has the potential to enhance patient safety, lessen effects of the learning curve, and prepare the next generation of spine surgeons for the expanding role of minimally invasive techniques.

## Figures and Tables

**Table 1:** Summarized results from analyzing the effectiveness of various training modalities in improving resident performance with MISS techniques.

Study	MISS Technique	Training Type	Primary Outcomes
Zaki et al. (2024) [19], World Neurosurgery	MIS-LLIF	VR lateral spine module	Increased precision scores and decreased radiograph usage for the majority of resident participants. Reduced operation time and increased confidence in performing MIS-LLIF in all participants
Akbulut et al. (2024) [20], Asian Spine Journal	MIS Lumbar Discectomy	3D-printed MIS spinal model	Mean operative time decreased from 21 min 18 s to 6 min 45 s after fourth practice with model (*p* < 0.0001)
Chitale et al. (2013) [21], Neurosurgery	Pedicle Screw Insertion	Didactic curriculum + MIS simulation model	Mean written test score improved from 78% to 100% after a 2 h didactic curriculum; Improvements in technical score for CT and fluoroscopic navigation also improved, although this was not statistically significant
Buchanan et al. (2019) [22], Operative Neurosurgery	MIS Dural Repair	Perfusion-based cadaveric model	Mean dural closure time improved from 12 min 7 s to 7 min 4 s (*p* = 0.02)
Schmidt et al. (2025) [23], Operative Neurosurgery	MIS-TLIF	High-Fidelity Lumbar Spine Simulation model ± Augmented Reality	AR supplementation resulted in significantly decreased mental demand (*p* = 0.003) and significantly less difficulty in maintaining performance levels during the procedure (*p* = 0.019)
Rambani et al. (2014) [24], Journal of Surgical Education	Pedicle Screw Insertion	Computer Simulation	Significant improvements in operative time, fixation accuracy, and reduction in fluoroscopy exposures (*p* < 0.05)
Harrop et al. (2013) [25], Neurosurgery	Navigation	Didactic curriculum + PCDF Simulation Model	Didactic scores improved in 78% of participants (*p* = 0.005); technical scores increased from a mean of 14.1 to 22.4 (*p* = 0.02).
Walker et al. (2009) [26], Neurosurgery	Pedicle Screw Insertion/MISS Laminectomy	Surgical simulator with animal model	Improvements in self-reported junior and senior resident confidence in MISS laminectomy and pedicle screw insertion, respectively
Stienen et al. (2014) [27], Acta Neurochirurgica	Microscopic Lumbar Disk Herniation repair	Resident involvement in surgery	No significant differences in intraoperative blood loss, surgery duration, complication rates, post-surgical pain reduction, or quality of life outcomes between teaching and non-teaching cases.
Ryu et al. (2017) [28], World Neurosurgery	Pedicle Screw Insertion	Computer-based simulator; synthetic model	Computer-based simulators successfully incorporate procedural guidance and real-time feedback, while synthetic models provide more realistic haptic feedback and allow for utilization of real surgical tools
Luciano et al. (2013) [29], Neurosurgery	Percutaneous Needle Placement	Mixed Augmented Reality + Haptic Feedback Simulator	Performance accuracy significantly improved between first and second attempts (*p* = 0.04)
Kirkpatrick (2012) [30], Journal of Spinal Disorders & Techniques	Pedicle Screw Insertion	Mentored surgical training on models	Mentored residents showed significantly greater improvements in performance scores (*p* = 0.0068). Subsequent screw placement error rate was significantly lower in the mentored group than non-mentored controls (*p* = 0.004)
Sundar et al. (2016) [31], Journal of Neurosurgery: Spine	Pedicle Screw Insertion	Cadaver or Sawbones model + surgical navigation training session	Significant reduction in overall surgical error (*p* = 0.04) compared to controls. Fewer errors in thoracic (*p* = 0.02) and lumbar (*p* = 0.04) regions, with more optimal screw placement in the cervical, thoracic, and lumbar regions (*p* = 0.02, *p* = 0.04, *p* = 0.04, respectively)
Gardeck et al. (2020) [32], Journal of Neurosurgery: Spine	Pedicle Screw Insertion	Lecture + Synthetic spine model w/3D computer-assisted navigation	Regardless of previous experience, all residents showed significant improvement on subjective measures for navigated screw placement (*p* < 0.001). Nearly all residents showed improvements on objective measures for navigated screw placement (*p* < 0.001) and reduced their screw placement time from session 1 to session 2 (*p* = 0.006)
Melcher et al. (2023) [33], Global Spine Journal	MIS-ULBD	High-Fidelity Simulator	By the third practice, the average procedural time decreased by 31.7 min, while skipped steps and surgical errors significantly declined. Surgical proficiency improved, particularly in efficiency, smoothness, and instrument handling. Knowledge gap decreased by 30% (*p* = 0.001), with the greatest gains among junior residents.
Alaraj et al. (2013) [34], Neurosurgery	Pedicle Screw Insertion	Multi-modal augmented reality simulator	Less fluoroscopy necessary to achieve accurate pedicle screw placement

## Data Availability

No new data was created or analyzed for this review.

## Supplementary Materials

The following supporting information can be downloaded at: https://www.mdpi.com/article/10.3390/brainsci15090936/s1, Table S1: Search terms for PubMed, Embase, and Scopus databases.

## Abbreviations

The following abbreviations are used in this manuscript:

MIS	Minimally Invasive Surgery
MISS	Minimally Invasive Spine Surgery
TLIF	Transforaminal Lumbar Interbody Fusion
LLIF	Lateral Lumbar Interbody Fusion
ULBD	Unilateral Laminotomy for Bilateral Decompression
PCDF	Posterior Cervical Decompression and Fusion
AR	Augmented Reality
VR	Virtual Reality
