# Supplementary material for: Resident Training in Minimally Invasive Spine Surgery: A Scoping Review
Source: Brain Sci. 2025 Aug 28;15(9):936. doi: 10.3390/brainsci15090936 (PMC12467833; doi:10.3390/brainsci15090936)
Supplement: Supplementary file 1 [file brainsci-15-00936-s001.zip › brainsci-3748525-supplementary.pdf]

Supplementary Table S1. Search terms for PubMed, Embase, and Scopus databases.

PubMed	(((((((("Minimally Invasive Surgical Procedures"[Mesh]) AND ("Neurosurgery"[Mesh] OR "Neurosurgical Procedures"[Mesh])) OR ("Orthopedics"[Mesh] OR "Orthopedic Procedures"[Mesh])) AND ("Education"[Mesh] OR "Postdoctoral Training"[Mesh] OR "High Fidelity Simulation Training"[Mesh] OR "Simulation Training"[Mesh] OR "Preceptorship"[Mesh])) OR "Computer Simulation"[Mesh]) OR "Learning Curve"[Mesh]) AND "Internship and Residency"[Mesh]) AND "Spine"[Mesh] NOT "Anesthesia, Spinal"[Mesh]) NOT "Ultrasonography"[Mesh]
Embase	('minimally invasive surgery'/exp OR 'minimally invasive surgery') AND ('neurosurgery'/exp OR 'neurosurgery' OR 'neurosurgical procedure'/exp OR 'neurosurgical procedure' OR 'orthopedic surgery'/exp OR 'orthopedic surgery' OR 'orthopedic procedure') AND ('education'/exp OR 'education' OR 'postgraduate education'/exp OR 'postgraduate education' OR 'simulation training'/exp OR 'simulation training' OR 'preceptorship'/exp OR 'preceptorship' OR 'computer simulation'/exp OR 'computer simulation' OR 'learning curve'/exp OR 'learning curve') AND ('internship'/exp OR 'internship' OR 'residency'/exp OR 'residency') AND ('spine'/exp OR 'spine') NOT ('anesthesia spinal'/exp OR 'anesthesia spinal') NOT ('ultrasonography'/exp OR 'ultrasonography')
Scopus	TITLE-ABS-KEY ((("minimally invasive surgery" OR "minimally invasive surgical procedures") AND ("neurosurgery" OR "neurosurgical procedures" OR "orthopedics" OR "orthopedic procedures") AND ("education" OR "postdoctoral training" OR "simulation training" OR "high fidelity simulation" OR "preceptorship" OR "computer simulation" OR "learning curve") AND ("internship" OR "residency" OR "graduate medical education") AND "spine") AND NOT ("spinal anesthesia" OR "ultrasonography"))
